# Omega-3 Fatty Acids as a Treatment for Pediatric Depression. A Phase III, 36 Weeks, Multi-Center, Double-Blind, Placebo-Controlled Randomized Superiority Study

**DOI:** 10.3389/fpsyt.2019.00863

**Published:** 2019-11-27

**Authors:** Isabelle Häberling, Gregor Berger, Klaus Schmeck, Ulrike Held, Susanne Walitza

**Affiliations:** ^1^Research Department of Child and Adolescent Psychiatry, University Hospital of Psychiatry Zurich of the University of Zurich, Zurich, Switzerland; ^2^Research Department of Child and Adolescent Psychiatry, Psychiatric University Hospitals Basel, University of Basel, Basel, Switzerland; ^3^Epidemiology, Biostatistics and Prevention Institute, University of Zurich, Zurich, Switzerland

**Keywords:** omega-3 fatty acids, clinical trial protocol, randomized controlled trial, pediatric depression, treatment of depression

## Abstract

**Background:** Depressive disorders in childhood and adolescence are a major health problem and often follow a chronic course with severe consequences in later life. Depressive disorders cause the highest burden of disease in this age group across all medical conditions. Treatment adherence is usually very poor, and the use of antidepressant drugs is heavily debated, as suicidal ideations may increase, in particular in the early phase of treatment. Omega-3 fatty acids rich in eicosapentaenoic acid have shown some promising results in over a dozen small scale randomized controlled trials (RCTs) in adult major depressive disorders, with only very few published RCTs in children and adolescents. High-quality phase III RCTs are missing.

**Methods and Design:** The omega-3-pMDD trial is a carefully designed phase III RCT to assess the efficacy and safety of omega-3 fatty acids in the early course of pediatric major depressive disorder (MDD). The study is designed as a multi-center, double-blinded, placebo-controlled, randomized clinical trial enrolling 220 patients aged 8 to 17 years meeting DSM-IV criteria for major depressive disorder of at least moderate symptom severity. After a single-blinded placebo-lead-in phase (7 to 10 days) patients are randomly assigned to omega-3 fatty acids or placebo over 36 weeks. Primary outcomes are changes in depression severity, as well as remission and recovery rates. Secondary outcome measures include the omega-3 index and inflammatory parameters as predictors of response. Data analysis will be performed in the intention-to-treat sample using a (generalized) linear random intercept regression model. Through sampling of blood, hair, saliva, and urine, further putative biological markers for depression and omega-3 fatty response will be investigated.

**Discussion:** This trial addresses if omega-3 fatty acids play a role in the pathogenesis of pediatric MDDs and have antidepressant properties, in particular in clinically depressed children and adolescents with a pre-existing omega-3 fatty acid deficiency, increased markers of oxidative stress, and/or markers of (low grade) inflammation.

**Ethics and Dissemination:** The study was approved by the local ethics committees. The results will be published in peer-reviewed journals irrespective of specific outcomes.

**Clinical Trial Registration:** www.ClinicalTrials.gov, identifier NCT03167307.

## Introduction

Prevalence rates of major depressive disorders (MDDs) are low in prepubertal children, but increase substantially throughout adolescence. In a recent representative survey of over 10,000 adolescents aged 13 to 18 years, lifetime and 12-month prevalence were estimated at 11.0% and 7.5%, respectively (1). Females have a three times higher risk than males to undergo a first depressive episode between the ages of 12 and 17 years (2). About 20% of childhood-onset MDD will recover within 3 months and about 60% within 9 months, which puts the mean length of childhood onset MDD to about 9 months (3). A more recent survey estimated the mean duration of an episode to be about 27 weeks (1), but with high individual variability (4). An early onset of the disease is a risk factor for chronic and recurrent forms of depression in adulthood with more than half experiencing a first relapse within five years (5). MDD is associated with difficulties in relationships, impaired school and work functioning, conduct problems, and an increased risk of substance abuse (2,, 7). Additionally, depressive disorders are major contributors to the burden of suicide and poor long-term health later in life (8, 9).

Depressive disorders are often not recognized by professionals (10) and only about a quarter receive appropriate treatment (11). These findings are alarming given that MDD is the leading cause of disability between 10 and 24 year olds (12).

### Omega-3 Fatty Acids and Depression

The dramatic change in human life style, in particular in dietary habits, might be one of the reasons for the raise of civilization diseases, such as obesity, cardiovascular diseases, cancer, inflammatory, and autoimmune diseases, but also mental disorders such as depression, given that in modern societies the nutritional environment does not match the human genetic constitution (13). The modern Western dietary habits have changed in an unprecedented way since the industrial revolution with a dramatic shift from a balanced omega-3 to omega-6 ratio towards an excessive intake of omega-6 fatty acids. Nowadays, there are hardly any processed foods without an excess of omega-6 fatty acids due to the widespread industrial use of vegetable oils (e.g. sunflower oil). Furthermore, the intake of fish and other sources of omega-3 long chained polyunsaturated fatty acids (LC-PUFAs) have decreased, in particular in urban areas.

Epidemiological studies provide some evidence that high intake of fish seems to be a protective factor against the development of MDD (14), in particular in females (15). Case-control studies confirm that a high fish intake reduces the relative risk for child and adolescent depression (16). Reduced levels of omega-3 fatty acids in red blood cells of depressed patients provide further evidence supporting a link between altered PUFA metabolism and depression (17). Interestingly, such a reduction in PUFAs is only found in patients experiencing a current depressive episode, but not in remitted patients (18, 19), suggesting a direct and reversible relationship between omega-3 fatty acids deficiency and depression.

Meta analyses summarizing the results of various randomized placebo-controlled clinical trials on the efficacy of omega-3 fatty acids treatment in MDD reported standardized mean differences ranging from 0.11 to 0.56 (an overview of all RCTs is given in **SI Table 1**), reaching different conclusions of the clinical significance of omega-3 fatty acids as an antidepressant (AD) treatment (20–30). A recent rigorous Cochrane review encompassing 20 RCTs in MDD suggests a small-to-modest benefit for depressive symptomology with an SMD = −0.32 (27). Some of the inconsistencies between studies may also be explained by methodological differences, such as the inclusion/exclusion criteria, study subgroup selection, and choice of drug composition. For example, omega-3 fatty acids have only been found to be effective in populations with a clinical diagnosis of depression, but not in subclinical depressed individuals (23, 28). In addition, formulations rich in eicosapentaenoic acid (EPA) seem to be more effective than formulations rich in docosahexaenoic acid (DHA) (26), although it is unclear whether the effects depend on a specific ratio of EPA to DHA (24) or solely on a higher EPA dose irrespective of the DHA content (28, 29). Furthermore, omega-3 fatty acids might be especially effective in depressed patients with no comorbid disorders (30), and some studies have shown that it is also effective as an augmentation to another AD therapy, possibly even more so (28, 29, 31). Nearly all meta-analyses come to the conclusion that, at present, we do not have sufficient evidence to support or rule out that omega-3 PUFAs are potential treatments for MDD or could reduce dosages of selective serotonin reuptake inhibitors (SSRI) medication in MDD. Adequately powered RCTs are, therefore, warranted.

### Evidence in Children and Adolescents

In contrast to the abundance of studies conducted in adults, the evidence of a link between omega-3 fatty acids and depression in children and adolescents is relatively sparse. In a Japanese study including 6,700 adolescents aged 12 to 15 years daily fish intake was inversely associated with depressive symptoms, but only in college boys and not girls (16). Red blood cell fatty acid levels have also been shown to be lower in depressed adolescents than in healthy controls (32) although the highest effects were found in other fatty acids than EPA or DHA.

The few randomized controlled trials conducted in minors showed at least partly encouraging results (see **SI Table 1**). In a small study including 20 depressed children aged 6 to 12 years, omega-3 fatty acid supplementation showed a large advantage over placebo (33). Given the small sample size, though, these results need to be interpreted with caution. Similarly, an RCT including 23 young adults around 20 years of age also showed significant reduction in depression severity after 3 weeks of omega-3 fatty acids treatment (34). In another small scale study including 35 patients with half of them getting a daily dose of 2,400 mg omega-3 fatty acids and the other half getting the same amount of omega-6 fatty acids (35), a significant treatment effect was found for patients with a major depressive disorder. Patients with a mixed anxiety depressive disorder, however, did not benefit from the omega-3 fatty acid treatment. The largest study to date included 72 youth with pediatric depression aged 7 to 14 years which underwent omega-3 monotherapy, individual-family psychoeducational psychotherapy (PEP) or combinations, respectively, for a trial duration of 12 weeks (36). Omega-3 treatment showed a small to medium effect compared to placebo. In an interesting study including 14 adolescents with SSRI resistant MDD, half of the patients received a very high dose of 16.2 g omega-3 fatty acids per day and the other half a low dose of 2.4 g per day over 10 weeks (37). In the high-dose group, 100% of patients showed symptoms remissions compared to 40% in the low dose group. In contrast though, no beneficial effects of omega-3 fatty acids were found in an RCT including 51 adolescents with MDD who were treated for a period of 10 weeks (38). In summary, preliminary data point towards beneficial effects of omega-3 fatty acids on pediatric depression but large-scale studies are needed to validate these results.

### Omega-3 Fatty Acids, Depression, and Cognition

Cognitive deficits are often observed in depressed individuals reflected in reduced concentration, attention, and impairments in executive and memory functions, among others (39). Even more so, residual cognitive deficits may persist after remission of the depressive episode affecting academic performance (40). The role of omega 3 fatty acids in improving cognitive functions has been extensively studied in a variety of populations ranging from infants to the elderly, and from healthy individuals to patients with psychiatric, neurodegenerative or neurodevelopment disorders. For example, omega 3 fatty acids supplementation has been shown to improve depressive symptoms and verbal fluency in elderly with mild cognitive impairments (41), although the effects might depend also on genetic variations of the patients (42). However, other studies reported no or only small beneficial effects (43, 44), or only on specific cognitive subdomains such as reaction time (45).

The evidence of a beneficial effect of omega-3 fatty acids on cognition in children and adolescents remains inconclusive. In a Danish study assessing the impact of a healthy diet on school performance in third and fourth grade children, the dietary intervention improved school performance and reading comprehension. Post hoc analysis revealed that about 20% of the intervention effect might be attributed to an increase in EPA and DHA levels due to the intake of fish (46). Similarly, end-term grades and vocabulary were found to be higher in the group of 700 Dutch high school students who met the national guidelines of fish consumption compared to the group who never ate fish (47). In healthy adolescents aged 13 to 15 years, the blood level ratio between omega-3 and omega-6 correlated with scores on the letter Digit Substitution Test, indicating a higher information processing speed (48).

A recent meta-analysis confirmed a beneficial effect of omega-3 fatty acids in infants up to 18 months of age but not in children or adults (49), although the inclusion of a wide range of assessment instruments and age stages might raise questions regarding the validity of the result (50). Even if omega-3 fatty acids might not improve cognition in healthy developing children, they might alleviate cognitive deficits in adolescents with psychiatric disorders. For example, omega-3 fatty acid supplementation for 16 weeks improved working memory but no other cognitive functions in children diagnosed with attention-deficit/hyperactivity disorder (ADHD) (51). Similarly, the level of DHA was lower in ADHD children with learning difficulties than in those without, and higher DHA levels predicted better word reading skills (52). In a recent meta-analysis Chang and colleagues (53) concluded that omega-3 fatty acids supplementation improves cognitive functioning associated with attention in ADHD children. Surprisingly, only one study to date assessed directly whether omega-3 fatty acid supplementation will not only improve mood symptoms in pediatric depression but also associated cognitive deficits. Vesco and colleagues (54) found improved executive functioning after a 12-week omega-3 fatty acid intervention. It remains to be established whether omega-3 fatty acids have beneficial effects on other cognitive domains that are often affected in pediatric depression, such as working memory or attention. Given the high prevalence of cognitive deficits in depressed youth and their impact on academic performance and school work, more studies using a set of different cognitive tests are necessary to evaluate differential effects of omega-3 fatty acids on cognitive functioning in depressed children and youth.

### Biological Mechanism

Preclinical and clinical data point toward several mechanisms that most likely act in concert (55). For example, omega-3 fatty acids have shown to attenuate the exaggerated, persistent elevation of the stress response in animal models with depressive features (56–58) and humans (59, 60). Several lines of evidence support an altered immune-modulation in the pathophysiology of depression. Inflammatory mediators, like cytokines, play an important role in the stress system by influencing the activity of the hypothalamic pituitary adrenal (HPA) axis (61). Chronic stress, elicits a neuroinflammatory response, releasing inflammatory mediators, such as interleukin-1β (IL-1β) and tumor necrosis factor-α (TNF-α) (62), two cytokines that are inhibited by omega-3 fatty acids. Chronic stress also alters activation of the immune system in the periphery, which might account for the state of chronic inflammation observed in depressed patients (63). Omega-6 fatty acids such as arachidonic acid (AA) and omega-3 fatty acids such as EPA and DHA are both converted in bioactive molecules, also called eicosanoids. The eicosanoids derived from omega-3 and omega-6 fatty acids have partially opposing functions, with the first ones being mainly pro-inflammatory and the latter ones acting anti-inflammatory (64, 65). Both compete with each other for incorporation into cell membrane phospholipids (66–68). Omega-3 fatty acids inhibit the release of pro-inflammatory cytokines, such as IL-1β, IL-2, IL-6, interferon-γ, and TNFα (69). Given the link between depression and an increased production of pro-inflammatory cytokines (70), this inhibition might explain some of the AD effects of omega-3 fatty acids. Furthermore, cytokines also lower neurotransmitter precursor availability, and neurotransmitter metabolism (71), which might be important in the pathogenesis of mood disorders.

Omega-3 fatty acids are also crucially important for brain development. Myelination and synaptic pruning are core processes during normal pubertal brain development (72). The regulation of PUFA metabolism is crucial for both processes (73, 74). Of particular interest is a preclinical study investigating cognition and behavior across different developmental stages in rats. Omega-3 fatty acids deficient diets across consecutive generations produced a modality-selective and task-dependent impairment in cognitive and motivated behavior in adolescent rats distinct from the deficits observed in adult rats (75, 76). Omega-3 fatty acids supplementation was able to attenuate such depression-like animal behaviors during critical periods of brain development (77). Furthermore, omega-3 fatty acids have a preventive and neurotrophic effect against structural hippocampal alterations in animal models with depression- and anxiety-like behaviors (78–81). In addition, supplementation with omega-3 fatty acids might increase synaptic plasticity through an increase of brain-derived neurotrophic factor (BDNF), acting neuroprotective (71). Omega-3 fatty acids might ameliorate symptoms of depression through the protection of cells and their positive effects on neurogenesis, and counteract the shrinkage of the hippocampus (82). Omega-3 fatty acids deprived rats also provide evidence for an increase in serotonin 2 (5-HT2) and a decrease in dopamine 2 (D2) receptor density in the frontal cortex, as well as an increased serotonin turnover in the prefrontal cortex and decreased midbrain tryptophan hydroxylase-2 expression (83–89). In humans, omega-3 intake is associated with an increase in cerebrospinal fluid 5-5-hydroxyindoleacetic acid (5-HIAA) release (90, 91).

Omega-3 fatty acids are essential components of intracellular und neuronal cell membranes, affecting cell membrane integrity and fluidity (69, 92, 93). Through their effect on the efficiency of membrane functioning, omega-3 fatty acids play a role in a variety of biological mechanisms, such as enzyme and receptor activity, ion channels functioning, and the production and activity of neurotransmitters (85, 94). An increase in membrane fluidity results in a more flexible membrane and facilitates transmission (95).

While supplementation of omega-3 fatty aims to increase concentrations of fatty acids and their derived bioactive lipids that are available to the organism, omega-3 fatty acid metabolism depends on a variety of factors, including genetic variations. Therefore, omega-3 fatty concentrations will be monitored over the course of the study with blood sampling procedures carried out at baseline, after 3 months, and at the end of the study. Remaining blood is stored in a biobank allowing for further analyses of various parameters associated with omega-3 fatty acid balance and depression as well as the impact of genetic makeups on fatty acid metabolism.

### The Omega-3-pMDD Trial: Rationale

As summarized above, there is growing evidence for the efficacy of omega-3 fatty acids as a treatment for major depression, in particular in pediatric populations. The generalizability of the results, though, is hampered by the conduction of small-scale studies, which do not allow for an accurate estimation of effect sizes. Therefore, the implementation of a well-designed large-scale clinical trial is warranted.

The present omega-3-pMDD trial recruits 220 patients with clinically diagnosed pediatric depression to assess the efficacy and safety of omega-3 fatty acids treatment. Given the knowledge gained from trials conducted in adults, formula rich in EPA will be used. In addition, comorbid disorders are assessed systematically for each patient, and concomitant medication is recorded throughout the 36-week trial. In case no concomitant medication is given, the efficacy of omega-3 fatty acid supplementation as a first line treatment is evaluated. However, ethical considerations do not allow for all patients to be treated solely with omega-3 fatty acids or placebo over a period of 36 weeks. In cases of severe or treatment resistant depression, additional pharmacological therapies might be indicated. In a recent survey, 40% of 85,000 depressed adolescents received AD medication during the course of their illness (96). This number appears relatively high given the major concern of increased suicidal ideations in the first weeks of treatment, which has been linked to the intake of ADs in minors (97, 98). In 2004, the advisory board of the Food and Drug Administration (FDA) decided to issue a black box warning indicating an increased risk of suicidal ideation in children receiving AD medication, especially SSRIs. Although further meta-analyses debated the increase of suicidal ideation after use of these drugs (99), it has contributed to a lot of insecurity in affected individuals and their families, as well as in the professional community (100).

When we designed the Omega-3 pMDD study, we evaluated several possible study designs. If we had decided to do a pure augmentation trial, the likelihood to lose those families opposed to AD medication (quite common in pediatric populations) would have been large. If we had decided to do a pure monotherapy omega-3 PUFAs RCT, we were concerned that clinicians would only refer less severe cases raising the risk of a ceiling effect due to the natural recovery rates of less severely depressed patients. Furthermore, omega-3 fatty acids seem to have better efficacy in severely depressed patients (23), potentially because more severe states of depression may be associated with stronger inflammatory processes. Both options were therefore considered less favorable to investigate the primary aim that omega-3 PUFAs have AD properties in moderately to severely depressed children and adolescents. For all these reasons, we decided that concomitant treatment with AD medication is permitted to not compromise the representativeness of the sample and to ensure that patients receive the optimal therapy for their disorder. Our design will allow us to directly compare the effects of omega-3 fatty acids as a monotherapy as well as adjunct to other AD drugs. In addition, statistical methods including multiple imputations in those who are put on ADs will be employed to control for the effects of AD medications on the trials’ results. Without controlling for AD use, we would run the risk to compare omega-3 fatty acids with ADs instead of placebo. In the proposed additional intention-to-treat analysis, we will consider the start of AD medication as a dropout criterion and impute the consecutive assessment time points of the main outcome measurements based on the values of those subjects that are not put on an AD. By considering the beginning of an AD as a treatment failure, we minimize the risk to mask a positive effect of omega-3 fatty acid supplementation by starting ADs more frequently or earlier in the placebo arm than in the omega-3 fatty acid arm. With this unique und novel way of analyzing the primary outcome data, the omega-3-pMDD trial aims to answer a wide range of questions regarding the treatment with omega-3 fatty acids, such as their efficacy and safety, the influence of omega-3 fatty acid supplementation on cognitive deficits associated with depression, and the emergence of putative biomarkers for omega-3 fatty acid treatment with and without the confounding effects of ADs.

## Methods and Analysis

### Study Overview

The Omega-3-pMDD study is a Swiss multicenter, randomized, double blind, placebo controlled clinical trial, enrolling a sample of 220 individuals aged 8 to 17 years who have a present primary diagnosis of a major depressive disorder of at least moderate symptom severity. The study design incorporates a 7- to 10-day lead in phase and a 36-week double blind placebo-controlled treatment phase. The study was approved by the local ethics committees and the regulatory affairs and is registered at ClinicalTrials.gov protocol No NCT03167307. The primary outcome is changes in the Children’s Depression Rating Scale (CDRS-R) total score (101, 102). The categorical co-primary outcome includes response rates at 6 weeks and remission rates at 12 and 36 weeks, and rates of recovery defined by the absence of major depression for > 4 months at 36 weeks. Secondary outcome measures are a variety of psychopathological, neuropsychological, and biological measures, including questionnaires, cognitive testing, and biological markers. In addition, potential response predictors are assessed, such as inflammatory mediators in serum, red blood cell acids, and bioactive lipid mediators.

### Interventions

#### Intervention Group

The active treatment consists of a daily dose of 1,000 mg EPA/500 mg DHA in the over 13 years old. Meta-analyses in adults have shown that only drugs containing an EPA content over 60% are effective in the treatment of depressive disorders (24). Furthermore, according to the meta-analysis conducted by Grosso and colleagues (103), the optimal daily dose of omega-3 fatty acids is about 1.8 g, which is very close to the 1.5 g administered in the current trial. In children under 13 years old, half the dose is administered resulting in 500-mg EPA/250 mg DHA per day, which is a similar daily dose to the one used in the trial of prepubertal children conducted by Nemets and colleagues (33).

#### Control Group

The control group receives placebo capsules containing mostly medium chain triglycerides (MCT), because they do not contain any unsaturated fatty acids, and in these small quantities, they do not have any pharmacological effect. A major concern is that omega-3 fatty acids capsules could be “unblinded” because of the fishy taste. Therefore, a small amount of fish oil is added to the placebo capsules to mimic the taste, preventing guessing of the treatment arm (104). Capsules are especially manufactured so that they can easily be swallowed by children. In addition, they will contain natural orange flavor for a pleasant odor when opening the bottle and so optimizing compliance.

### Concomitant Interventions

The background treatment across both groups is standardized based on the evidence and consensus-based German S3 Guidelines for the treatment of depression in children and adolescents (105). The core element is based on the cognitive behavioral therapy (CBT) method that includes individual CBT as well as psycho-educational family sessions. All participant sites are trained accordingly. In severe or treatment resistant cases of depression, the prescription of AD drugs might be indicated by experienced senior clinicians of the participating centers. Medicated patients can still participate in the trial. However, the start time and dose of the AD medication are recorded and are taken into account in the statistical analyses, as described further below.

### Participants and Recruitment Methods

#### Participant Eligibility

**Table 1** displays the inclusion and exclusion criteria used in the trial and their corresponding rationale. Given that omega-3 fatty acids were shown to be mainly efficient in clinically diagnosed patients (23), diagnosis criteria for major depressive disorder are carefully assessed to ensure that all patients fulfill the diagnosis of a major depressive disorder (MDD) according to DSM-IV. In addition, the severity of the symptoms needs to be of at least moderate severity, as measured by a revised Children’s Depression Rating Scale (CDRS-R) total score of ≥40. Patients with the primary diagnosis of schizophrenia, bipolar affective disorder, substance use dependency, pervasive developmental disorders (autism), neurological conditions or the diagnosis of an eating disorder within the last 6 months are excluded from the trial. Other comorbid disorders, such as attention deficit disorders, anxiety disorder or conduct disorders, are not considered to be a reason for exclusion of the trial, but are recorded as pre-specified comorbidities to allow the identification of clinical subgroups which might differ in their treatment response. Furthermore, routine laboratory blood testing is performed before the intervention time to ensure the well-being of the patients and to detect any medical problems. Given the ongoing debate whether omega-3 fatty intake might increase bleeding time (106), coagulation parameters will be assessed and in case of clinically significant values patients will not be able to take part in the trial.

**Table 1:** Inclusion and exclusion criteria Omega-3-pMDD trial.

*Inclusion Criteria*	*Rationale*
*Exclusion Criteria*	*Rationale*
Male or female in- or outpatients of a participating center	Standard treatment of depression according to S3 guidelines
Youth aged 8 to 17 years	Matches developmental sensitivity of treatment and measures
Primary diagnosis of major depressive disorder with depressive symptoms of at least moderate severity (CDRS-R total score of ≥40)	Clinically significant and diagnosed MDD
No clinically significant laboratory findings.	Guarantees safety and proper treatment
Able to swallow the study medication without difficulty	Guarantees compliance
Contraindications to the class of drugs under study, e.g. known hypersensitivity or allergy	Guarantees safety of patients (e.g. fish allergy)
Regular omega-3 supplementation within the last 6 months	Potential confounding factor
Lack of safe contraception; women who are pregnant, breast feeding, or have the intention to become pregnant during the course of the study	Ethical considerations
Pre-existing neurological (such as brain tumor, temporal lobe epilepsy, HIV encephalopathy) or medical conditions likely to be responsible for the depressive symptoms	May require additional or different treatment
Known or suspected non-compliance	Guarantees compliance
Inability to follow the procedures of the study, e.g. due to language problems, psychological disorders, dementia, etc. of the participant	Cannot complete study assessment
Participation in another study with investigational drug within the 30 days preceding and during the present study	Potentially confounding factor
Previous enrolment into the current study	Potentially confounding factor
Enrolment of the investigator, his/her family members, employees, and other dependent persons	Ethical considerations
Substance dependency (ICD-10 F1x.2) within the last 6 months (but not misuse)	May require different or additional treatment
Lifetime diagnosis of schizophrenia and related disorders (ICD-10 F20-F25)	May require different or additional treatment
Lifetime diagnosis of bipolar affective disorder in the K-SADS-PL (ICD-10 F30, F31)	May require different or additional treatment
Current eating disorders within the last 6 months (ICD-10 F50.0 and F50.2),	May require different or additional treatment, compliance risk
Mental retardation (ICD-10 F70-73)	May require different or additional treatment, difficulties following research assessments
Pervasive development disorder (ICD-10 F84.x)	May require different or additional treatment

#### Recruitment

Children and adolescents meeting criteria for clinical depression are recruited from mental health in- and outpatient services in the German speaking part of Switzerland. In all participating centers, the study team performs annual workshops introducing the study. Patients and their families are only contacted directly by the investigators once clinicians have explored their willingness to be informed about the study. Thereafter, the first visit is scheduled during which patients and their families are informed about the nature and course of the study, and the study information and informed consent are handed out. The study team answers all questions, and the families are advised not to sign immediately but to think it over for at least another day before enrolling. Once the patients and their legal guardian have signed, the consent form appointments for the first week are scheduled.

Research assessments are carried out by the central study team, which will visit patients and their families in the participating centers or at their homes. All research psychologists will complete training courses for the clinical interviews and the cognitive tests prior to visiting patients. Scoring of clinical interviews will be tested using video footage of patients being assessed. Furthermore, all new staff members will be directly observed in their first research assessments, and symptoms will be scored simultaneously by other researchers until a good agreement is reached. To ensure the reliability of the data and to detect any inconsistencies in assessments, weekly research meetings will be held, during which symptom ratings and fulfillment of diagnostic criteria are discussed with senior clinicians (GB, SW).

### Stratification and Randomization

The data are stratified by sex (male, female), age group (≥ 13 years old, < 13 years old), center, and whether the patient belongs to an in- or outpatient unit. In addition, the value of the high sensitive C-reactive protein (hsCRP) forms another stratification parameter (low < 1 mg/L; average 1.0–3.0 mg/L; high > 3.0 mg/L). Previous studies in adults (107, 108) and adolescents (109) have shown serum hsCRP to be an independent risk factor in depressive disorders, highlighting the role of low grade inflammation in the pathogenesis of mood disorders. Given the anti-inflammatory mechanisms of omega-3 fatty acids, a balanced allocation of patients with and without low-grade inflammation in the treatment arms are warranted. Randomization is carried out using the in-built functions of the clinical trial software SecuTrial^®^. SecuTrial^®^ assigns the patient to the subgroup which leads to least imbalance within all strata. The software then provides a randomization number from a list implemented previously by a data administrator not otherwise involved in the study. Given that not always the next randomization number is selected by the computer, the study team is unable to guess what the next number will be. The research psychologist hands out the correct trial medication, which are stored in identical bottles and with the number as only identification.

### Assessments

#### Primary Outcome Assessments/Parameters

The primary outcome is change in the total score of the Children’s Depression Rating Scale- revised (CDRS-R), measured on a continuous scale. Co-primary categorical outcomes are response rate to treatment at week 6, remission rate at weeks 12, 24, and 36, and recovery rate at 36 weeks. The CDRS-R is a 17-item scale and quantifies childhood (102) and adolescent (110) depressive symptoms with a total score of ≥40 indicating moderate to severe depression and has been extensively used in research (111). The scale is also evaluated in German (112). Response is defined as a 30% decrease in the total CDRS-R scores (113), whereas a score ≤28 is being used to define remission. Recovery is defined as the absence of the diagnosis of MDD for > 4 months, and is established with the semi-structured interview Kiddie Schedule for Affective Disorders and Schizophrenia — Present and Lifetime Version (K-SADS-PL) (114). The K-SADS-PL is a diagnostic interview designed to assess current and past DSM-IV diagnoses in children and adolescents, by interviewing the parent(s) and child. The section for diagnosing the presence of a current major depressive disorder confirms the presence of a MDD at baseline and determines recovery status of a study participant at each assessment time point. In addition, the K-SADS-PL is also used to assess comorbid disorders and to ensure inclusion and exclusion criteria are met.

#### Secondary Outcome Assessments/Parameters

Secondary outcome assessments consist of a variety of questionnaires, rating scales, and neuropsychological tests, as summarized in **Table 2**. Depression severity is additionally assessed through a self-report questionnaire, the Depression Inventory for Children and Adolescents (DIKJ) (115). Suicidal ideation is assessed using the Suicidal Ideation Questionnaire (118) with adolescents over 13 years also filling in the Beck’s hopelessness Scale (116). Anxiety levels are assessed through the Beck’s anxiety inventory (117), and emotional instability as well as other borderline traits are rated by parents and patients over 13 years of age using the German versions of the IES-27 (124). Stress is monitored with the Perceived Stress Scale (PSS) (121), while the Connor Davidson Resilience Scale (120) is used to estimate resilience. Sleep quality is assessed using the Insomnia Severity Index (119). Parents and patients rate quality of life with the Kidscreen (132) with parents and patients over the age of 11 years also rating behavioral problems using the Strength and Difficulty Questionnaire (SDQ) (123). In addition, childhood maltreatment is assessed with the Childhood Trauma Questionnaire (CTQ) (138) while victimization in the school environment is assessed with the Olweus Bullying Questionnaire (OBQ) (139).

**Table 2:** Outcome measures Omega-3-pMDD trial.

Primary outcome measures	Scale	Examiner
Secondary outcome measures (part A)		
Secondary outcome measures (part B)		
Clinician rated variables		
Cognitive outcome variables		
Biological outcome measures		
Other measures		
Depression severity	Children’s Depression Rating Scale (102)	Interview with parents and patients
Diagnosis of MDD	K-SADS-PL(114)	Interview with parents and patients
Depression severity	Children’s Depression Inventory (DIKJ) (115)	Self-report
Hopelessness	Beck Hopelessness Scale (116)	Self-report
Anxiety	Beck Anxiety Inventory (BAI) (117)	Self-report
Suicidality	Suicidal ideation questionnaire (SIQ-JR) (118)	Self-report
Sleep	Insomnia Severity Index (ISI) (119)	Self-report
Resilience	Connor-Davidson Resilience Scale (120)	Self-report
Stress	Perceived Stress Scale (PSS-10) (121)	Self-report
Quality of life	KIDScreen-Cat (122)	Self- and parent report
Behavior	Strength- and difficulties Questionnaire (SDQ) (123)	Self- and parent report
Emotional Instability	IES-27 (124)	Self- and parent report
Global impression	Clinical Global Impression Scale (125)	Clinician
Functioning	Children Global Assessment Scale (126)	Clinician
General health and social functioning	HoNOSCA (127)	Clinician
attrition	Attrition checklist	Clinician
Executive functions	BRIEF (128)	Self- and parent report
Memory	VLMT (129)	Researcher
Working memory	WISC-IV (130)	Researcher
Attention	ANT (131)	Researcher
Emotion recognition	ANT (131)	Researcher
Verbal fluidity	RWFT (132)	Researcher
Intelligence	Reynolds Intellectual Assessment Scales (133)	Researcher
Clinical blood	Electrolytes, red and white blood tests (e.g. hemoglobin, hematocrit, leucocytes), thyroid function test, liver, and kidney function test, thrombocytes, thromboplastin (blood coagulation)	Study nurse (processed and analyzed in a certified clinical laboratory)
Blood	Omega-3, 6,9 fatty acid levels and bioactive lipids (e.g. eicosanoids)	Study nurse (centrifuged immediately after blood taking; further processing in a specialized research laboratory of the children’s hospital Zürich)
Urine	Drug screen, F2 isoprostane	Study nurse
Saliva	Cortisol	Study nurse
hair	Steroid profile	Study nurse
Side effects	ASEC (134)	Self-report
Compliance	MARS-D (135)	Self-report
Compliance	Pill count	Researcher
Omega-3 intake	Omega-3 Food Frequency Questionnaire (136)	Self-report
Puberty status	Tanner questionnaire (137)	Self-report/parents for younger children
Childhood maltreatment	Childhood Trauma Questionnaire (138)	Self-report
Bullying/victimization	Olweus Bully/Victim Questionnaire (139)	Self-report
Sociodemographic data	Questionnaire	Interview, clinic information system

#### Clinician-Rated Scales

In addition to self- and parent-rated instruments, clinicians treating the patients are asked to fill in three short rating scales. Clinicians rate symptom severity and global improvement on the Clinical Global impression scale (CGI-S/I) (125) and global functioning on the Children’s Global Assessment of functioning (CGAS) scale (126). In addition, behavior, impairment, symptom severity, and social functioning are scored on the Health of the Nation Outcome Scale for Children and Adolescents (HoNOSCA) (127). Furthermore, clinicians indicate how many minutes they spent treating the patient between study assessments.

#### Cognitive Assessments

The level of cognitive functioning is assessed using a variety of standardized neuropsychological tests. Different episodic memory parameters are assessed with the Verbal Memory Learning Test (VMLT) (129), while working memory is tested with the Digit Span Forward and Backward of the Wechsler Intelligence Test for Children (WISC IV) (130). The Regensburger Word Fluency Test measures verbal fluency as a function of divergent thinking (132). The ability to shift attention and inhibit responses is assessed with the *Shifting Visual Attention* Test of the computerized Amsterdam Neuropsychological Test battery (131). Included in the same test battery is also the *Identifying Facial Emotions* test which assesses the ability of a subject to recognize emotions displayed in faces. In addition, executive functioning is assessed with the Behavior Rating Inventory of Executive Function (BRIEF) (128, 140), in which parents and patients over the age of 11 years rate the level of impairment of executive functioning in daily life. And finally, intellectual abilities are assessed with the Reynolds Intellectual Assessment Scale (RIAS) (133).

#### Biological Assessments

Blood is drawn three times during the course of the study, at baseline, 12 weeks, and at 36 weeks, respectively. At baseline, routine laboratory parameters are assessed to ensure that the patients are healthy and do not need any immediate medical attention. The remaining blood is stored for PUFAs (omega-3, -6, and -9 and trans fatty acids), bioactive lipids (e.g., eicosanoids, hydroxy metabolites, E-series resolvins), immune parameters (including but not restricted to interferon-γ, IL-1α, IL-1RA, IL-5, Il-6, IL-10, IL12p40, IL-15, IL-18, and TNF-α, as well as leptin and adiponetin), and for the tissue repository. Since multiple studies are currently investigating biomarkers in MDD, serum, lymphocytes, erythrocyte, platelets, and DNA will be stored after the end of the study to measure potential novel biomarkers. Genetic and epigenetic markers of interest (e.g. FADS haplotypes) include but are not restricted to genes relevant for bioactive lipid metabolism. In addition to the blood sample, patients are asked to provide 3 cm of hair in order to assess the steroid profile, including cortisol, testosterone, progesterone, dehydroepiandrosterone, and estradiol levels at 6 weeks and 9 months. Furthermore, saliva (cortisol, melatonin) and urine (drug screen, F2 isoprostane as a marker of lipid oxidation) are sampled. Biological samples are stored in a biobank for use in future research if patients and their parents agree to sign an additional consent form.

#### Compliance, Safety, and Other Measures

Compliance is monitored with the Medication Adherence Reporting Scale (MARS-D) (135). In addition, returned capsules are counted at each study visit. Potential side effects are assessed with a modified version of the Antidepressant Side Effect Checklist (ASEC) at each visit (134). The daily nutritional intake of omega-3 fatty acid is estimated with an adapted version of the Omega-3 Food Frequency Questionnaire (136), three times during the course of the trial.

### Study Schedule

**Figure 1:**
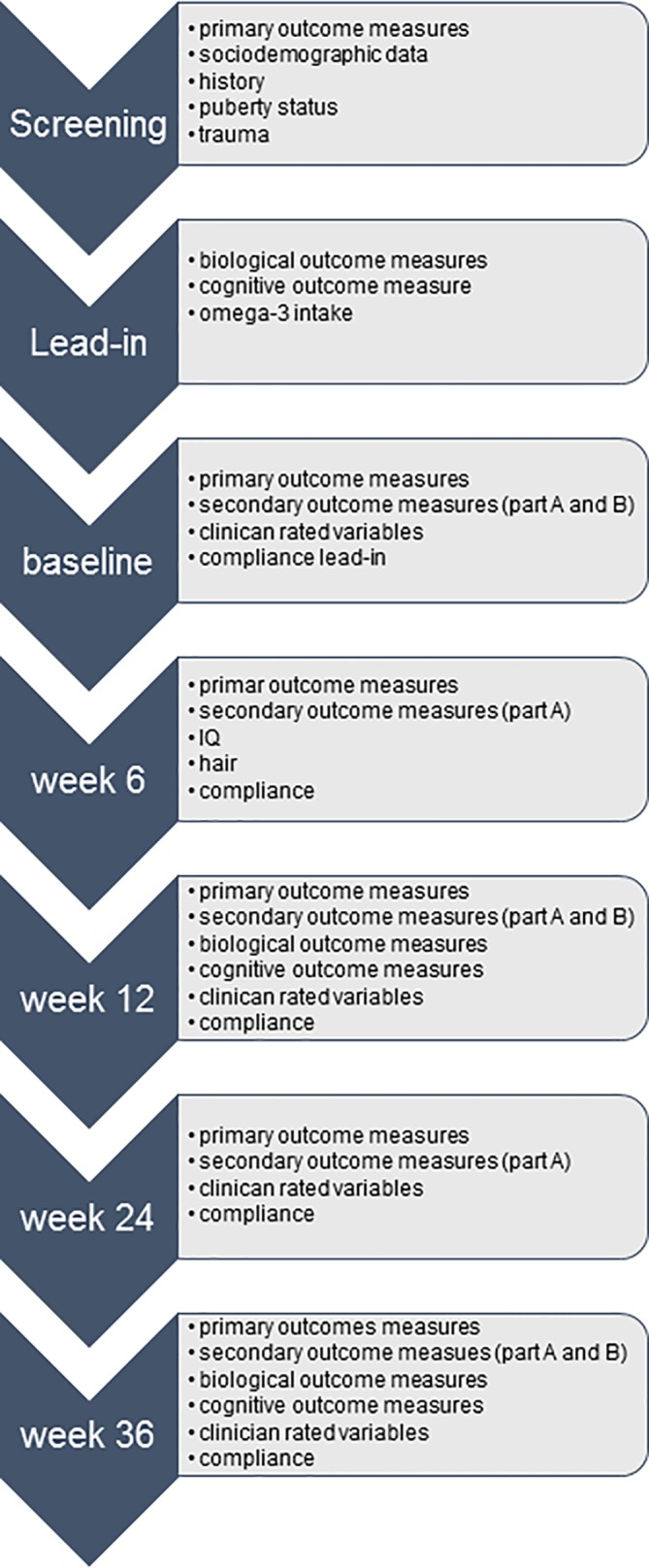
Flowchart study schedule Omega-3-pMDD trial.

A flowchart of the study schedule is depicted in **Figure 1**. After the consent process, patients and their families undergo a screening interview in order to assess inclusion criteria, such as the diagnosis and severity of the depressive disorder and exclusion criteria, such as comorbidities that might prevent participation. Sociodemographic data and information about medical and family history and current medication use is also collected. If a patient is considered eligible for the trial, he or she enters the 7 to 10 days single blinded placebo lead-in phase. All patients receive placebo capsules for at least 7 days prior to randomization in order to assess any difficulties in swallowing the medication and measure compliance. Furthermore, the lead-in phase allows the identification of fast placebo responders. The baseline visit takes place at the end of the placebo lead-in phase, during which the CDRS and the subscale depressive disorder of the K-SADS-PL will be administered for a second time. If the patient still fulfills the diagnosis of a major depressive disorder with at least moderate severity, and no other exclusion criteria have emerged, the patient is randomized. Four follow-up assessments at 6, 12, 24, and 36 weeks are performed, consisting mainly out of the same measurements taken during the lead-in phase. Only four follow-up measurements are scheduled given that the number of research assessments has been identified as a predictor for placebo response (141, 142). The timing of the assessments was based on the design of the “Treatment for Adolescents with Depression” study (143), allowing us to make direct comparisons of illness trajectories between the trials. Intelligence is assessed only once at the 6-week appointment, and hair sample are taken at the 6-week appointment and at the end of the trial. Unused study drugs are counted at each appointment with compliance rates below 60% resulting in the discontinuation of the trial.

#### Participants’ Compensation

There is only a small compensation for participation with patients receiving a voucher of about 20 CHF three times during the course of the study. Additional travelling costs (i.e., train fares) are recompensed.

### Data Management and Analytic Approach

#### Data Acquisition and Security

All data collected for the Omega-3-pMDD study are entered into electronic case report forms hosted on a secure server of the Clinical Trials Centre of the University Hospital of Zurich. Each participant is assigned a study ID at enrolment, which is subsequently used to enter data into the clinical trial software SecuTrial^®^ (www.secutrial.com). All data that are not entered directly by the patients or their parents are entered twice by two different investigators with another investigator checking the consistency of the double data entry and resolving all conflict. In addition, data quality is ensured through regular on-site monitoring by the Clinical Trials Centre of the University Hospital of Zürich. Data security, on the other hand, is guaranteed by encrypted communication and stringent firewall settings. Automated backups are made every 24 h to guarantee that no data are lost. Confidentiality of the data will be granted at all times.

#### Sample Size

Our power calculation was based on the available meta-analysis at the time of grant submission. The available meta-analyses provided an expected SMD of omega-3 PUFAs between 0.28 and 0.56 mainly based on adult omega-3 intervention trials (22, 24–26,). However, at this time point no studies in pediatric MDD were published, except one very small (n = 20) pilot study in childhood depression showing a large effect size (SMD = 1.4) (33). Because of the confounding factor of concomitant AD therapy and an expected high placebo response in pediatric populations, we introduced a range of measures, such as a placebo-lead in phase and conservative expected difference effect size between active and placebo. We used the following assumptions for our power calculation: Cohen’s d as effect size, calculated as difference between means in the two treatment groups divided by their pooled standard deviation, was considered to be 0.4, and with a significance level alpha of 5% and a power of 80%, 100 patients per group would be necessary to detect the clinically relevant difference with a two-sample, two-sided t-test, resulting in 200 patients in total. With an anticipated dropout rate of 10% within the first six weeks after randomization (the time point of the first follow up assessment), the number of patients for this study would be 220. The study is sufficiently powered to address also the co-primary endpoint according to the following considerations: the proportion of patients with clinically relevant improvement in the group of patients receiving omega-3 is P(omega-3) = 0.60, and the corresponding proportion of patients in the placebo group is P(placebo) = 0.40. With a significance level of 0.05 for a two-tailed chi-squared test and an anticipated power of 80%, 97 patients per treatment group would be needed. With an anticipated dropout rate of 10%, 220 patients would be sufficient to address this clinically relevant endpoint.

Based on the preliminary analysis of the baseline data of the on-going trial, 2/3 of the patients are AD free at the time of randomization, with most of them still being AD free after the first follow-up. A sample size of n = 74 per treatment arm in this subgroup of AD-free participants will enable us to detect a moderate SMD of 0.54 using a two-group *t* test for the CRSR-R total score with a 0.05 two-sided significance level and a 90% power. The entire ITT sample (also encompassing the third with pre-existing AD treatment) will even enable us to detect small effect sizes between active/placebo. Meta-analyses of omega- 3 PUFA supplementation in depression suggest that omega-3 PUFA augmentation trials show an even larger effect size compared to omega-3 monotherapy RCTs (31, 144, 145), in particular if EPA rich formulations or pure EPA is considered (146).

### Statistical Analysis

Descriptive statistics include mean and standard deviation for continuous variables, as well as number and percentages of total for categorical variables. Ordinal scaled variables will be reported as median and interquartile range. Descriptive statistics will be reported within treatment groups.

Primary analysis is performed based on the intention to treat (ITT) principle, i.e. including all randomized patients. For the continuous primary outcome (total CDRS-R score), we will apply a linear random intercept regression model. For the co-primary endpoint, i.e., the binary outcome measure for treatment response, we will apply a logistic regression model, and treatment group as independent variable will be used. Results will be presented as effect estimates — beta-coefficients or odds ratios — with 95% confidence intervals for primary and co-primary endpoints, respectively.

However, the analysis of treatment efficacy of the omega-3 fatty acids needs to address that some study participants already receive ADs prior to study inclusion or will be put on ADs in the due course of the study. The additional use of ADs might not be uniformly distributed between the two treatment arms, which might introduce biased results if not accounted for. To account for this major confounding factor, we propose to use two different analytic approaches. (1) We will perform an additional ITT analysis of the primary and secondary outcomes defining the beginning of an AD as a treatment failure and include treatment failure/additional use of AD as time-dependent covariate in the random effects model. (2) Using 20-fold multiple imputation, depression scores that a patient would have had without AD prescriptions are estimated, based on consecutive data of subjects without AD, and on earlier observations in the same subjects. To address that the missingness generating mechanism is considered to be “missing not at random” (MNAR), the “drawn indicator method” proposed by Jolani et al. (147) will be applied. A subsequent sensitivity analysis of the results will reveal the dependence of the results on specific assumptions in the two different analytic approaches.

A pre-specified subgroup analysis will address whether there is a differential treatment effect between patients with or without the pre-specified variable (e.g. AD therapy at baseline). If there is evidence for an interaction (as quantified with the p-value of the corresponding interaction test), the treatment effect will also be reported in those subgroups.

Furthermore, we’ll run following analyses to investigate the impact of AD use based on *a priori* working hypotheses: Firstly, we will test differences in novel AD prescriptions between the treatment groups by means of the two proportions z-Test using the ITT sample (assuming that ADs are more frequently prescribed in the placebo arm). We will also calculate retention rates between the two groups by means of the two proportions z-Test prior of “dropping out” those subjects put on ADs (assuming that the retention rate is longer in the active compared to the placebo arm).

Further analyses on primary and secondary outcome measures will be run as detailed in the study protocol, which is available on the clinical.trials.gov website.

### Safety Considerations

Omega-3 fatty acids are usually well-tolerated, but according to a recent meta-analysis by Chang et al. (148), they might elicit mild adverse effects such as gastrointestinal symptoms in some patients. In a meta-analysis including 148 omega-3 fatty acid studies with dosage up to 6 g per day, 6.6% of the active group relative to 4.3% in the placebo group reported gastrointestinal complaints. Only one study reported an increase in bleeding time (64). Thus, any medical conditions experienced during the trial are recorded. In addition, patients fill in the ASEC at each visit to measure other putative side effects. No doses changes of active/placebo are permitted during the trial, but patients and their families are informed of the voluntary nature of the study and that consent can be rescinded anytime. While no serious side effects are expected due to omega-3 fatty acids intake, the current patient population warrants for a tight safety control due to the risk of suicide. Therefore, suicidal tendencies will be closely monitored by the study team by asking directly about suicidal ideations during the clinical interviews in addition to the administration of the Suicidal Ideation Questionnaire (SIQ-Jr). Furthermore, a data monitoring committee (DMC) is set up consisting of an expert in child and adolescent psychiatry, an expert in ethical considerations, and an expert in biostatistics. All members have access to the unblinded data due to permissions associated with their study function in the database SecuTrial. In case of a serious adverse event, the members of the DMC are automatically notified through an inbuilt function of the software in order for them to continuously monitor the safety of the patients. Furthermore, after 60 enrolled patients, an interims analysis on the safety of the treatment is was performed by a member of the DMC.

### Publication and Dissemination

The main outcome of the trial is published irrespective of the results. A publications committee establishes publication guidelines and coordinates the publication of secondary outcome measures, also giving recommendations to the timing of different abstracts, reviewing all publications for their appropriateness and scientific merit. No later than five years after closure of the data base, a completely de-identified data set will be provided for sharing purposes.

## Discussion

This paper describes the design and methodology for a clinical trial evaluating the efficacy and safety of omega-3 fatty acids as a treatment for pediatric depression. It is paramount for children and adolescents suffering from mental problems to receive appropriate treatment. However, affected families often don’t seek help because of the stigma associated with mental illness and psychiatric treatments (149). Being open to novel treatments such as omega-3 fatty acids that are closely linked to lifestyle, food, and general well-being may indirectly encourage affected families to seek help sooner. If omega-3 fatty acids prove to be beneficial, they might serve as a benign well-accepted first step in a comprehensive treatment plan. On the other hand, if omega-3 fatty acids prove to be ineffective, the results are as important given the risks associated with delaying other effective treatments due to an unjustified supplementation of omega-3 fatty acids. Therefore, this well-designed large-scale clinical trial will provide important information on the optimal management and treatment of pediatric depression.

In our study design, we permit concomitant AD medication, to ensure the representativeness of our sample and the inclusion of more severely depressed patients. However, we will study the confounding effects by running subgroup analyses and statistically controlling for use of AD drugs. We believe that the abovementioned measures and the large sample size will allow us to address both the question if omega-3 fatty acids are effective as a first-line treatment, as well as to address the question of augmenting a pre-existing AD treatment with omega-3 PUFAs will further improve treatment outcomes.

The long study duration of 36 weeks allows studying the trajectory of a variety of psychopathological and cognitive symptoms associated with depression. Patients fill in a full range of questionnaires ranging from anxiety and emotional instability to sleep disturbances^1^ to dietary omega-3 fatty intake. This allows the characterization of symptoms that are affected by omega-3 fatty acid intake and the establishment of subgroups of patients profiting the most from the treatment. Pre-treatment blood levels of omega-3, 6, and 9 fatty acids are used to predict treatment response, and a variety of biological parameters are assessed in order to enhance our understanding of the biological mechanism associated with depression and the effects of omega-3 fatty acids on the brain. This is especially important given the on-going brain development in minors and the severe impacts of pediatric depression on brain structure and function (150). The establishment of a biobank creates a database of a highly unique sample that allows for further studying of the underlying biological mechanisms of pediatric depression. Therefore, this trial will advance our understanding of the pathogenesis of depression in minors and will provide a valuable contribution to the best treatment options of this severely affected patient group.

## The Omega-3 Study Team

The Omega-3 Study Team contributed with implementation of the design with following roles: Sponsorinvestigator of the trial is GB (Department of Child and Adolescent Psychiatry, University Hospital of Psychiatry, University of Zurich, Neumünsterallee 9, 8032 Zurich, Switzerland; gregor. berger@puk.zh.ch; +41 43 499 2626). Chief investigators are SW and KS. IH is study coordinator. Principal investigators and research psychologist from the clinical sites are as follows: Principal Investigator Zurich: SW; Research psychologists: Noemi Baumgartner, Sophie Emery, Mona Albermann, and Kristin Nalani (Department of Child and Adolescent Psychiatry, University Hospital of Zurich); Principal Investigator Basel: KS; Investigators and research psychologists: Oliver Pick, Alain Di Gallo, and Michael Strumberger (Department of Child and Adolescent Psychiatry, Psychiatric University Hospitals Basel); Principal Investigator Basel-Stadt: Brigitte Contin; Investigator: Stefan Müller (Child and Adolescent Psychiatric Services Baselland); Principal Investigator: Silke Bachmann; Investigators: Lars Wöckel, and Simone Heitzer (Clienia Littenheid); Principal Investigator: Bruno Rhiner; Investigators: Amir Yamini (Child and Adolescent Psychiatric Services Thurgau); Principal Investigator: Suzanne Erb; Investigators: Michael Schmid (Child and Adolescent Psychiatric Services St. Gallen); Principal Investigator: Ulrich Müller-Knapp; Investigator: Ioannis Christodoulakis (Klinik Sonnenhof). UH and Burkhardt Seifert (retired) are statistical consultants. Renate Drechsler is head of the neuropsychology department and Edna Grünblatt head of the department for translational molecular psychiatry (Department of Child and Adolescent Psychiatry, University Hospital of Zurich). Martin Hersberger is head of the division of Clinical Chemistry and Biochemistry at the University Children’s Hospital Zürichand his PhD student Ivan Hartling of the division of Clinical Chemistry and Biochemistry who will analyze the bioactive lipids; Romuald Brunner (University of Heidelberg), Jürgen Drewe (University of Basel), and Julia Braun (Epidemiology, Biostatistics, and Prevention Institute, University of Zürich) are members of the Data Monitoring Committee. Jenny Peterson, Clinical Trials Pharmacy (Kantonsapotheke) Zürich, responsible for the packaging, handling, and quality of the study medication.

## Author Contributions

GB conceived of the study. GB, SW, KS, and IH devised the study design. UH lent statistical support.

## Funding

This research was funded as an investigator initiated clinical trial by the Swiss National Foundation (SNF grant 33IC30_166826). Furthermore, the Thalmann Foundation (University of Basel) supports an embedded sleep add on project, the Ebnet Foundation supports an additional PhD project in collaboration with the ETH Zürich investigating the influence of diet on depression and treatment response to omega-3 fatty acids. The Research Department of Child and Adolescent Psychiatry, University Hospital of Psychiatry Zurich of the University of Zurich further provides infrastructure and administrative staff support. Burgerstein, Antistress AG, Rapperswil-Jona (SG, Switzerland) provided the study medication (active and placebo). No additional industrial funding is provided throughout the study.

## Conflict of Interest

The company that provided the study medication (Burgerstein, Antistress AG, Rapperswil-Jona (SG, Switzerland) provides no direct financial support towards the RCT. Burgerstein, Antistress AG has no influence on the design or analysis of the trial or publication of any results in relation to the study. The lead authors declare following conflict of interests: IH: no conflict of interest; GB: GB was supported by the Swiss National Science Foundation, the Stanley Foundation, the Gertrud Thalmann Fonds, and the Ebnet Foundation and has received speaker honoraria from Lundbeck, Opopharma, Antistress AG (Burgerstein) in the last 5 years; KS: In the last 5 years KS has received royalties from Springer, Hogrefe, and Schattauer. Since 2014, KS received no honoraria from pharmaceutical or other industrial companies. His work was supported in the last 5 years by the Swiss National Science Foundation (SNF), Swiss Ministry of Justice, University of St. Gallen, Botnar Foundation, and Gertrud Thalmann Fonds. UH: no conflict of interest. SW: SW has received in the last 5 years royalties from Thieme Hogrefe, Kohlhammer, Springer, Beltz. SW received lecture honoraria from Opopharma in the last 5 years. Her work was supported in the last 5 years by the Swiss National Science Foundation (SNF), diff. EU FP7s, HSM Hochspezialisierte Medizin of the Kanton Zurich, Switzerland, Bfarm Germany, ZInEP, Hartmann Müller Stiftung, Olga Mayenfisch, Gertrud Thalmann Fonds. Outside professional activities and interests are declared under the link of the University of Zurich.

## Footnotes

1 An add-on study under supervision of Prof. Christian Cajochen and Prof. Klaus Schmeck will investigate the effects of omega-3 fatty acids supplementation on sleep and physical activity in a subgroup of the depressed patients.
