# Supplementary material for: Omega-3 Fatty Acids as a Treatment for Pediatric Depression. A Phase III, 36 Weeks, Multi-Center, Double-Blind, Placebo-Controlled Randomized Superiority Study
Source: Front Psychiatry. 2019 Nov 27;10:863. doi: 10.3389/fpsyt.2019.00863 (PMC6892434; doi:10.3389/fpsyt.2019.00863)
Supplement: Supplementary file 1 [file Table_1.pdf]

Supplementary table 1: published randomized control trials on the efficacy of omega-3 fatty acids in depression.

Publication year	Study[1]	N[2] (intervention/ control)	Mean age	Study duration (weeks)	Rating	Antidepres-sants allowed	Indication	EPA dose (g/ day)	DHA dose (g/day)	Results	Conclusion
	Pediatric depression										
**2018**	Gabbay et al.	48 (21/27)	16	10	CDRS; BDI	no	adolescent MDD	flexible 0.8 - 2.4	flexible 0.4 - 1.2	-	No difference in CDRS or BDI scores between the groups.
**2017**	Trebaticka et al.	35 (17/18)	15	12	CDI	yes	pMDD pMADD	1	0.75	+	Higher reduction of depressive scores in the omega-3 compared to an omega-6 group. Subgroup analysis revealed positive effects of omega-3 in pMDD but not pMADD.
**2016**	Fristad et al.	69 (17/18/16/ 18)	12	12	CDRS	no	pMDD	1.4	0.2	+	Small to medium effects for combined treatment and omega-3 monotherapy, but no effect for PEP and placebo or placebo alone.
**2014**	McNamara et al.	17 (7/10)	16	10	CDRS	yes	SSRI-resistant adolescent MDD patients	low 1.6; high 10.8	low 0.8; high 5.4	+	Symptom remission in 100% of high dose group and 40% of low dose group (no placebo group).
**2006**	Nemets et al.	20 (10/10)	10	16	CDRS	no	pMDD	0.4	0.2	+	Higher reduction of depressive symptoms in the omega-3 compared to the placebo group.
	Adult depression										
**2019**	Carney et al.	144 (73/71)	59	10	BDI; HAMD	yes augmentation	MDD with high risk for coronary heart disease	2	0	-	No difference in depression scores between EPA and placebo group, augmentation to sertraline
**2019**	Chang et al.	59 (30/29)	61	12	HAMD, BDI	no	MDD and coronary heart disease	2	1	-	No difference in depression severity with exception of the HAMD cognition subscale between the groups; effect of omega-3 fatty acids only in the very severe depression group.
**2019**	Sarris et al.	158 (81/77)	42	8	MADRS	yes	MDD	1	0.656	-	No differences in depression scores between omega-3 and placebo group.
**2019**	Tayama et al.	79 (42/37)	40	12	BDI	unclear	Mild to moderate depression	1.06	0.56	-	No difference in depression scores between the groups.
**2018**	Jiang et al.	80 (28/24/28)	58	12	HAMD	SSRI allowed	MDD and CHF	2 or 1.6	0 or 800	-	No differences in depression scores between the groups.

**2018**	Keshavarz et al.	45 (24/21)	42	12	BDI	no	MDD and obesity	1.08	0.72	+	Reduction of depressive symptoms and increased weight loss in the omega-3 fatty acids compared to the placebo group.
**2018**	Su et al.	27 (14/13)	45	12	HAMD, BDI	no	MDD	3.5 or 0	0 or 1.75	+	EPA group higher decrease in HAMD scores than DHA group (no placebo group).
**2016**	Masoumi et al.	60 (30/30)	55	4	BDI	yes, augmentation	Post-menopausal depression	?	?	+	Lower depression scores in the omega-3 group compared to placebo, augmentation to citalopram.
**2016**	Shinto et al.	31 (15/16)	51	12	MADRS	yes	MDD with MS	1.95	1.35	-	No differences between omega-3 and placebo group.
**2015**	Mischoulon et al.	177 (60/58/59)	46	8	HAMD, QIDS, CGI	no	MDD	1.06 or 0.18	0.24 or 0.9	-	No differences between EPA, DHA or placebo groups on any depression scale.
**2015**	Park et al.	25 (12/13)	42	12	CES-D-K, HAMD, CGI,	yes	MDD	1.14	0.6	+	Omega-3 fatty acid group showed higher reduction of the CGI scores but not on CES-D-K or HAMD scores compared to placebo.
**2013**	Mozaffari-Khosravi et al.	62 (21/20/21)	35	12	HAMD	yes	Mild to moderate depression	1	1	+	Higher reductions of HAMD scores in the EPA group compared to the DHA and placebo group.
**2012**	Gertsik et al.	32 (17/15)	41	8	HAMD, BDI, MADRS	yes, augmentation	MDD	0.9	0.2	+	Significantly greater improvement in the omega-3 + citalopram group compared to the placebo + citalopram group on the HAMD scores, trend for BDI and MADRS scores.
**2011**	Lespérance et al.	432 (218/214)	46	8	IDS, MADRS	yes	MDD	1.05	0.15	+	Higher symptom reduction in the omega-3 group for the MDD patients without comorbid anxiety disorders compared to the placebo group.
**2011**	Tajalizadekhoob et al.	61 (32/29)	79	24	GDS	yes	Elderly depression	0.18	0.12	+	Higher reduction in GDS scores in the omega-3 compared to the placebo group.
**2010**	Rondanelli et al.	46 (22/24)	84	8	GDS	no	Geriatric MDD	1.67	0.83	+	Higher response and remission rates in the omega-3 compared to the placebo group.
**2010**	Bot et al.	25 (13/12)	54	12	MADRS	yes	MDD and Diabetes mellitus	1	0	-	No difference between omega-3 and placebo group.
**2009**	Carney et al.	122 (62/60)	58	10	BDI, HAMD	Yes, augmentation	MDD and coronary heart disease	0.93	0.75	-	No difference between omega-3 and placebo group.
**2009**	Mischoulon et al.	35 (16/19)	42	8	HAMD	no	MDD	1	0	-	No differences between omega-3 and placebo group, trend for study completers according to protocol.
**2008**	Rogers et al.	190 (96/94)	38	12	DASS, BDI,	no	Mild to moderate depression	0.63	0.85	-	No differences between omega-3 and placebo group.

**2008**	Da Silva et al.	29 (14/15)	64	12	MADRS, BDI, CGI	Yes	MDD and Parkinson disease	0.72	0.48	+	Higher reduction in depressive symptoms in the MADRS and CGI, but not the BDI, in the omega-3 compared to the placebo group.
**2008**	Su et al.	24 (13/11)	31	8	HAMD, EPDS, BDI	no	MDD; pregnant women	2.2	1.2	+	Higher reductions in all depression scales in the omega-3 compared to the placebo group.
**2008**	Jazayeri et al.	48 (16/16/16)	35	8	HAMD	yes, direct comparison	MDD	1	0	+	EPA and Fluoxetine combined were superior compared to the usage of only one of the two.
**2007**	Grenyer et al.	60 (32/28)	45	16	HAMD, BDI	yes	MDD	0.6	2.2	-	No difference between omega-3 and placebo group.
**2005**	Silvers et al.	59 (29/30)	39	12	HDRS-SF, BDI	yes	Depressive Episode	0.6	2.4	-	No difference between omega-3 and placebo group.
**2003**	Su et al.	22 (12/10)	39	8	HAMD	yes	MDD	4.4	2.2	+	Higher reduction in HAMD scores in the omega-3 compared to the placebo group after 4 weeks of treatment.
**2003**	Marangell et al.	35 (18/17)	47	6	MADRS, HAMD	no	MDD	0	2	-	No difference in depressive symptoms between DHA and placebo group.
**2002**	Nemets et al.	20 (10/10)	53	4	HAMD	yes	MDD	2	0	+	Higher reductions in the HAMD scores in the omega-3 compared to the placebo group.
**2002**	Peet et al.	60 (15/16/15/14)	45	12	HAMD, MADRS, BDI	yes	Depressive episode	1, 2, or 4	0	+	All depression scales improved in the low dose group (1-g/d) compared to the placebo group, but not in the high dose groups.

BDI = Beck Depression Inventory; CDI = Children's Depression Inventory; CDRS = Children's Depression Rating Scale; CES-D-K = Centre for Epidemiological Studies Depression Scale Korean version; CGI = Clinical Global Impression; CHF = Chronic Heart Failure; DASS = Depression, Anxiety and Stress Scales; DHA = docosahexaenoic acid; E-EPA = Ethyl eicosapentaenoic acid; EPA = eicosapentaenoic acid; EPDS = Edinburgh Postnatal Depression Scale; GDS = Geriatric Depression Scale; HAMD = Hamilton Rating Scale for Depression; HDRS-SF = Hamilton Depression Rating Scale – Short Form; IDS = Inventory of Depressive Symptomatology; MADRS = Montgomery-Asperg Depression Scale; MDD = major depressive disorder, MS = multiple sclerosis; PEP = Psychoeducational Psychotherapy; pMAD = pediatric mixed anxiety and depressive disorder; pMDD = pediatric major depressive disorder; QIDS = Quick Inventory of Depressive Symptomatology

[1] only one study per clinical trial is listed; [2] number of analysed subjects

References

Bot, M., Pouwer, F., Assies, J., Jansen, E.H.J.M., Diamant, M., Snoek, F.J., et al. (2010). Eicosapentaenoic acid as an add-on to antidepressant medication for co-morbid major depression in patients with diabetes mellitus: A randomized, double-blind placebo-controlled study. *Journal of Affective Disorders* 126(1-2)**,** 282-286. doi: 10.1016/j.jad.2010.04.008.

Carney, R.M., Freedland, K.E., Rubin, E.H., Rich, M.W., Steinmeyer, B.C., and Harris, W.S. (2009). Omega-3 augmentation of sertraline in treatment of depression in patients with coronary heart disease: A randomized controlled trial. *Jama-Journal of the American Medical Association* 302(15)**,** 1651-1657. doi: 10.1001/jama.2009.1487.

Carney, R.M., Freedland, K.E., Rubin, E.H., Rich, M.W., Steinmeyer, B.C., and Harris, W.S (2019). A randomized placebo-controlled trial of omega-3 and sertraline in depressed patients with or at risk for coronary heart disease. *J Clin Psychiatry* 80(4). doi: 10.4088/JCP.19m12742.

Chang, J.P., Chang, S., Yang, H.T., Chen, H.T., Chien, Y.C., Yang, B., et al. (2019). Omega-3 polyunsaturated fatty acids in cardiovascular diseases comorbid major depressive disorder - Results from a randomized controlled trial. *Brain, Behavior, and Immunity*. doi: 10.1016/j.bbi.2019.03.012.

da Silva, T.M., Munhoz, R.P., Alvarez, C., Naliwaiko, K., Kiss, A., Andreatini, R., et al. (2008). Depression in Parkinson's disease: A double-blind, randomized, placebo-controlled pilot study of omega-3 fatty-acid supplementation. *Journal of Affective Disorders* 111(2-3)**,** 351-359. doi: 10.1016/j.jad.2008.03.008.

Fristad, M.A., Vesco, A.T., Young, A.S., Healy, K.Z., Nader, E.S., Gardner, W., et al. (2016). Pilot randomized controlled trial of omega-3 and individual-family psychoeducational psychotherapy for children and adolescents with depression. *J Clin Child Adolesc Psychol***,** 1-14. doi: 10.1080/15374416.2016.1233500.

Gabbay, V., Freed, R.D., Alonso, C.M., Senger, S., Stadterman, J., Davison, B.A., et al. (2018). A double-blind placebo-controlled trial of omega-3 fatty acids as a monotherapy for adolescent depression. *Journal of Clinical Psychiatry* 79(4). doi: 10.4088/JCP.17m11596.

Gertsik, L., Poland, R.E., Bresee, C., and Rapaport, M.H. (2012). Omega-3 fatty acid augmentation of citalopram treatment for patients with major depressive disorder. *Journal of Clinical Psychopharmacology* 32(1)**,** 61-64. doi: 10.1097/JCP.0b013e31823f3b5f.

Grenyer, B.F.S., Crowe, T., Meyer, B., Owen, A.J., Grigonis-Deane, E.M., Caputi, P., et al. (2007). Fish oil supplementation in the treatment of major depression: A randomised double-blind placebo-controlled trial. *Progress in Neuro-Psychopharmacology & Biological Psychiatry* 31(7)**,** 1393-1396. doi: 10.1016/j.pnpbp.2007.06.004.

Jazayeri, S., Tehrani-Doost, M., Keshavarz, S.A., Hosseini, M., Djazayery, A., Amini, H., et al. (2008). Comparison of therapeutic effects of omega-3 fatty acid eicosapentaenoic acid and fluoxetine, separately and in combination, in major depressive disorder. *Aust N Z J Psychiatry* 42(3)**,** 192-198. doi: 10.1080/00048670701827275.

Jiang, W., Whellan, D.J., Adams, K.F., Babyak, M.A., Boyle, S.H., Wilson, J.L., et al. (2018). Long-chain omega-3 fatty acid supplements in depressed heart failure patients: Results of the OCEAN trial. *Jacc-Heart Failure* 6(10)**,** 833-843. doi: 10.1016/j.jchf.2018.03.011.

Keshavarz, S.A., Mostafavi, S.A., Akhondzadeh, S., Mohammadi, M.R., Hosseini, S., Eshraghian, M.R., et al. (2018). Omega-3 supplementation effects on body weight and depression among dieter women with co-morbidity of depression and obesity compared with the placebo: A randomized clinical trial. *Clinical Nutrition Espen* 25**,** 37-43. doi: 10.1016/j.clnesp.2018.03.001.

Lesperance, F., Frasure-Smith, N., St-Andre, E., Turecki, G., Lesperance, P., and Wisniewski, S.R. (2011). The efficacy of omega-3 supplementation for major depression: A randomized controlled trial. *Journal of Clinical Psychiatry* 72(8)**,** 1054-1062. doi: 10.4088/JCP.10m05966blu.

Marangell, L.B., Martinez, J.M., Zboyan, H.A., Kertz, B., Kim, H.F.S., and Puryear, L.J. (2003). A double-blind, placebo-controlled study of the omega-3 fatty acid docosahexaenoic acid in the treatment of major depression. *American Journal of Psychiatry* 160(5)**,** 996-998. doi: 10.1176/appi.ajp.160.5.996.

Masoumi, S.Z., Kazemi, F., Tavakolian, S., Rahimi, A., Oshvandi, K., Soltanian, A., et al. (2016). Effect of citalopram in combination with omega-3 on depression in post-menopausal women: A triple blind randomized controlled trial. *Journal of Clinical and Diagnostic Research* 10(10)**,** Qc1-Qc5. doi: 10.7860/Jcdr/2016/19487.8597.

McNamara, R.K., Strimpfel, J., Jandacek, R., Rider, T., Tso, P., Welge, J.A., et al. (2014). Detection and treatment of long-chain omega-3 fatty acid deficiency in adolescents with SSRI-resistant major depressive disorder. *PharmaNutrition* 2(2)**,** 38-46. doi: 10.1016/j.phanu.2014.02.002.

Mischoulon, D., Nierenberg, A.A., Schettler, P.J., Kinkead, B.L., Fehling, K., Martinson, M.A., et al. (2015). A double-blind, randomized controlled clinical trial comparing eicosapentaenoic acid versus docosahexaenoic acid for depression. *Journal of Clinical Psychiatry* 76(1)**,** 54-61. doi: 10.4088/JCP.14m08986.

Mischoulon, D., Papakostas, G.I., Dording, C.M., Farabaugh, A.H., Sonawalla, S.B., Agoston, A.M., et al. (2009). A double-blind, randomized controlled trial of ethyl-eicosapentaenoate for major depressive disorder. *Journal of Clinical Psychiatry* 70(12)**,** 1636-1644. doi: 10.4088/JCP.08m04603.

Mozaffari-Khosravi, H., Yassini-Ardakani, M., Karamati, M., and Shariati-Bafghi, S.E. (2013). Eicosapentaenoic acid versus docosahexaenoic acid in mild-to-moderate depression: a randomized, double-blind, placebo-controlled trial. *Eur Neuropsychopharmacol* 23(7)**,** 636-644. doi: 10.1016/j.euroneuro.2012.08.003.

Nemets, B., Stahl, Z., and Belmaker, R.H. (2002). Addition of omega-3 fatty acid to maintenance medication treatment for recurrent unipolar depressive disorder. *American Journal of Psychiatry* 159(3)**,** 477-479. doi: 10.1176/appi.ajp.159.3.477.

Nemets, H., Nemets, B., Apter, A., Bracha, Z., and Belmaker, R.H. (2006). Omega-3 treatment of childhood depression: a controlled, double-blind pilot study. *Am J Psychiatry* 163(6)**,** 1098-1100. doi: 10.1176/ajp.2006.163.6.1098.

Park, Y., Park, Y.S., Kim, S.H., Oh, D.H., and Park, Y.C. (2015). Supplementation of n-3 polyunsaturated fatty acids for major depressive disorder: A randomized, double-blind, 12-week, placebo-controlled trial in Korea. *Annals of Nutrition and Metabolism* 66(2-3)**,** 141-148. doi: 10.1159/000377640.

Peet, M., and Horrobin, D.F. (2002). A dose-ranging study of the, effects of ethyl-eicosapentaenoate in patients with ongoing depression despite apparently adequate treatment with standard drugs. *Archives of General Psychiatry* 59(10)**,** 913-919. doi: 10.1001/archpsyc.59.10.913.

Rogers, P.J., Appleton, K.M., Kessler, D., Peters, T.J., Gunnell, D., Hayward, R.C., et al. (2008). No effect of n-3 long-chain polyunsaturated fatty acid (EPA and DHA) supplementation on depressed mood and cognitive function: a randomised controlled trial. *The British journal of nutrition* 99(2)**,** 421-431. doi: 10.1017/S0007114507801097.

Rondanelli, M., Giacosa, A., Opizzi, A., Pelucchi, C., La Vecchia, C., Montorfano, G., et al. (2010). Effect of omega-3 fatty acids supplementation on depressive symptoms and on health-related quality of life in the treatment of elderly women with depression: A double-blind, placebo-controlled, randomized clinical trial. *Journal of the American College of Nutrition* 29(1)**,** 55-64. doi: 10.1080/07315724.2010.10719817.

Sarris, J., Byrne, G.J., Stough, C., Bousman, C., Mischoulon, D., Murphy, J., et al. (2019). Nutraceuticals for major depressive disorder- more is not merrier: An 8-week double-blind, randomised, controlled trial. *Journal of Affective Disorders* 245**,** 1007-1015. doi: 10.1016/j.jad.2018.11.092.

Shinto, L., Marracci, G., Mohr, D.C., Bumgarner, L., Murchison, C., Senders, A., et al. (2016). Omega-3 Fatty Acids for Depression in Multiple Sclerosis: A Randomized Pilot Study. *Plos One* 11(1). doi: 10.1371/journal.pone.0147195.

Silvers, K.M., Woolley, C.C., Hamilton, F.C., Watts, P.M., and Watson, R.A. (2005). Randomised double-blind placebo-controlled trial of fish oil in the treatment of depression. *Prostaglandins Leukotrienes and Essential Fatty Acids* 72(3)**,** 211-218. doi: 10.1016/j.plefa.2004.11.004.

Su, K.P., Huang, S.Y., Chiu, C.C., and Shen, W.W. (2003). Omega-3 fatty acids in major depressive disorder - A preliminary double-blind, placebo-controlled trial. *European Neuropsychopharmacology* 13(4)**,** 267-271. doi: 10.1016/S0924-977x(03)00032-4.

Su, K.P., Huang, S.Y., Chiu, T.H., Huang, K.C., Huang, C.L., Chang, H.C., et al. (2008). Omega-3 fatty acids for major depressive disorder during pyegnancy: Results from a randomized, double-blind, placebo-controlled trial. *Journal of Clinical Psychiatry* 69(4)**,** 644-651. doi: 10.4088/JCP.v69n0418.

Su, K.P., Yang, H.T., Chang, J.P.C., Shih, Y.H., Guu, T.W., Kumaran, S.S., et al. (2018). Eicosapentaenoic and docosahexaenoic acids have different effects on peripheral phospholipase A2 gene expressions in acute depressed patients. *Progress in Neuro-Psychopharmacology & Biological Psychiatry* 80**,** 227-233. doi: 10.1016/j.pnpbp.2017.06.020.

Tajalizadekhoob, Y., Sharifi, F., Fakhrzadeh, H., Mirarefin, M., Ghaderpanahi, M., Badamchizade, Z., et al. (2011). The effect of low-dose omega 3 fatty acids on the treatment of mild to moderate depression in the elderly: a double-blind, randomized, placebo-controlled study. *European Archives of Psychiatry and Clinical Neuroscience* 261(8)**,** 539-549. doi: 10.1007/s00406-011-0191-9.

Tayama, J., Ogawa, S., Nakaya, N., Sone, T., Hamaguchi, T., Takeoka, A., et al. (2019). Omega-3 polyunsaturated fatty acids and psychological intervention for workers with mild to moderate depression: A double-blind randomized controlled trial. *Journal of Affective Disorders* 245**,** 364-370. doi: 10.1016/j.jad.2018.11.039.

Trebaticka, J., Hradecna, Z., Bohmer, F., Vavakova, M., Waczulikova, I., Garaiova, I., et al. (2017). Emulsified omega-3 fatty-acids modulate the symptoms of depressive disorder in children and adolescents: a pilot study. *Child Adolesc Psychiatry Ment Health* 11**,** 30. doi: 10.1186/s13034-017-0167-2.
